# A multicenter phase II trial of anti-EGFR-immunoliposomes loaded with doxorubicin in patients with advanced triple negative breast cancer

**DOI:** 10.1038/s41598-023-30950-z

**Published:** 2023-03-06

**Authors:** Christoph Mamot, Andreas Wicki, Ursula Hasler-Strub, Salome Riniker, Qiyu Li, Lisa Holer, Daniela Bärtschi, Khalil Zaman, Roger von Moos, Konstantin J. Dedes, Laura A. Boos, Urban Novak, Alexandre Bodmer, Reto Ritschard, Ellen C. Obermann, Alexandar Tzankov, Christoph Ackermann, Véronique Membrez-Antonioli, Ursina Zürrer-Härdi, Clemens B. Caspar, Stefanie Deuster, Martin Senn, Ralph Winterhalder, Christoph Rochlitz

**Affiliations:** 1 Cantonal Hospital Aarau, Tellstrasse 25, 5001 Aarau, Switzerland; 2 University and University Hospital Zurich, Rämistrasse 100, 8091 Zürich, Switzerland; 3 Cantonal Hospital Graubünden, Chur, Switzerland; 4 Tumor and Breast Center East, St. Gallen, Switzerland; 5 Competence Center of the Swiss Group for Clinical Cancer Research (SAKK), Bern, Switzerland; 6 University Hospital Lausanne, Lausanne, Switzerland; 7 Breast Center Zürichsee, Zurich, Switzerland; 8 Inselspital, Bern University Hospital, University of Bern, Bern, Switzerland; 9 University Hospital Geneva, Geneva, Switzerland; 10 University Hospital Basel, Basel, Switzerland; 11 Spital STS AG, Thun, Switzerland; 12 Hôpital du Valais, Hôpital de Sion, Sion, Switzerland; 13 Cantonal Hospital Winterthur, Winterthur, Switzerland; 14 Cantonal Hospital Baden, Baden, Switzerland; 15 Hospital Lucerne, Lucerne, Switzerland

**Keywords:** Breast cancer, Drug development

## Abstract

Advanced triple negative breast cancer (TNBC) is an aggressive, but initially chemo-sensitive disease. The prognosis is poor and more than three quarters of patients experience progression 12 months after the initiation of conventional first-line chemotherapy. Approximately two thirds of TNBC express epidermal growth factor receptor 1 (EGFR). We have developed an anti-EGFR targeted nanocontainer drug by inserting anti-EGFR antibody fragments into the membrane of pegylated liposomes (anti-EGFR-ILs-dox). The payload consists of doxorubicin, a standard drug for TNBC. In a first-in-human phase I trial in 26 patients with various advanced solid malignancies, anti-EGFR-ILs-dox has shown little toxicity and encouraging efficacy. In this single-arm phase II trial, we assessed the efficacy of anti-EGFR-ILs-dox as first-line therapy in patients with advanced, EGFR + TNBC. The primary endpoint was progression-free survival at 12 months (PFS12m). Secondary endpoints included overall response rate (ORR), duration of response (DOR), time to progression (TTP), overall survival (OS) and adverse events (AEs). 48 patients received anti-EGFR-ILs-dox 50 mg/m^2^ iv, on day one of a 28 days-cycle until progression. The Kaplan–Meier estimate for PFS12m was 13% (one-sided 90% CI 7%, 95% CI [5%, 25%]), median PFS was 3.5 months (95% CI 1.9, 5.4). The trial has not reached its primary endpoint. There were no new toxicity signals. Based on these results, anti-EGFR-ILs-dox should not be further developed for TNBC. It remains an open question whether anti-EGFR-ILs-dox would offer more opportunities in other EGFR-expressing malignancies, where targeting this receptor has already shown anticancer effects.

Trial registration: This trial was registered at clinicaltrials.gov: NCT02833766. Registered 14/07/2016.

## Introduction

Breast cancer (BC) is the most frequently diagnosed cancer and the leading cause of cancer death in women worldwide, accounting for 25% of all new cancer cases and 15.5% of cancer deaths^1^. Triple negative breast cancer (TNBC), defined by the lack of expression of the estrogen receptor (ER), the progesterone receptor (PR), and the human epidermal growth factor receptor 2 (HER2), comprises approximately 15% of all BC and is associated with the worst prognosis of all subtypes. Unlike for other breast cancer subtypes, targeted treatment options are limited for this entity and no targeted agent is currently approved in the first line treatment setting.

Immunotherapy (in combination with chemotherapy) is available for those with advanced TNBC that express PD-L1 and has shown a significant overall survival benefit in patients with CPS ≥ 10%^2–4^. However, the impact of checkpoint inhibitors (anti-PD1/PDL1 antibodies) on survival of the overall population of patients with TNBC is modest. Classic chemotherapeutic agents, such as taxanes, anthracyclines and platinum-agents are the backbone of the first-line treatment of TNBC patients. Although response is frequent, TNBC relapse rapidly, and over time resistance to chemotherapy emerges^5–7^. The only approved targeted drug in a later line treatment setting is sacituzumab govitecan, an antibody–drug conjugate, which led to improved survival in a recent phase III trial^8^. Overall, treatment options remain limited and long-term remissions are unfortunately not seen with TNBC. Thus, there is a great demand for novel and improved therapeutic strategies in this patient group.

Liposomal encapsulation of drugs, in particular doxorubicin, is used to alter the tissue distribution and pharmacokinetics of the drug, increasing its therapeutic index by making use of the hyperpermeability of vascular structures in the proximity of neoplastic tissue. In clinical settings, pegylated liposomal doxorubicin has shown efficacy, first in the treatment of Kaposi's sarcoma^9^, and subsequently in metastatic or recurrent BC, ovarian cancer, multiple myeloma and other malignancies. In patients with metastatic BC, objective response rates (ORR) ranging from 5 to 33% have been reported^10^.

In order to achieve better efficacy and reduce toxicity, more specific delivery of liposomal doxorubicin to the target cells would be desirable. Up to 70% of TNBC express the epidermal growth factor receptor (EGFR), a tyrosine kinase receptor of the ErbB family, which is abnormally activated in many epithelial tumors^11^, thus making EGFR a promising target for specific delivery of anti-cancer drugs. EGFR can be targeted by cetuximab, an EGFR-binding monoclonal antibody (mAb), which is an established treatment option in patients with colorectal and head-and-neck cancer. Unfortunately, the use of anti-EGFR-directed mAbs in BC, either alone or in combination with chemotherapy, has shown disappointing results and, thus, does not play a role in clinical routine^12,13^.

While blocking the EGF receptor by a mAb does not lead to clinical benefit so far, we hypothesized that EGFR remains a valid target for drug delivery systems in breast cancer, due to the high frequency and expression levels of EGFR on the surface of TNBC tumor cells. Therefore, we constructed anti-EGFR targeted immunoliposomes by covalently inserting cetuximab fragments into the lipid layer of pegylated liposomal doxorubicin, creating a stable and robust drug carrier with a ligand, a mAb fragment, directing the encapsulated doxorubicin towards the target. In a series of preclinical experiments, we have shown that anti-EGFR targeted immunoliposomes may enhance drug efficacy in vivo^14^ and, perhaps even more importantly, overcome multidrug-resistance in EGFR-overexpressing cancer cells, including in the human breast cancer cell line MDA-MB-231^15^).

Based on these findings, we performed a classic dose finding phase I trial with this nanocontainer in patients with EGFR-expressing solid tumors. Toxicity was low and there were signs of clinical activity in heavily pretreated patients^4,16^. In the current trial, SAKK 24/14, we focused this approach on patients with advanced TNBC. TNBC is a suitable entity for targeted delivery of anti-EGFR–Ils-dox: (i) EGFR is often and robustly overexpressed in TNBC, (ii) in vitro studies have provided evidence of high efficacy of anti-EGFR-ILs-dox in EGFR overexpressing cancer cell lines^14,15^, and (iii) anthracyclines and doxorubicin in particular are widely accepted and active agents for first line palliative therapy in this cancer entity. Furthermore, this group of patients has (iv) a high clinical need for treatment improvements due to limited treatment options and prognosis despite of ongoing advances in the field.

The main objective of this trial, SAKK 24/14, is to determine the efficacy of anti-EGFR-ILs-dox as a first-line therapy option in this patient population.

## Methods

### Study sites and patient selection

This prospective, proof-of concept, open-label multicenter phase II trial (SAKK 24/14; NCT02833766) was designed with a single-stage single-arm layout. We accrued patients at 12 participating sites across Switzerland.

Patients met inclusion criteria if they presented with metastatic or locally advanced, inoperable TNBC with an EGFR expression of at least (1 +) on immunohistochemistry according to the EGFR pharmDx™ Interpretation Guidelines^17^. Staining was conducted with the ready-to-use K1492 EGFR pharmDx™ kit for manual use after antigen retrieval for 24 min with the CC1 buffer of Ventana/Roche and incubation for 32 min in the automated immunostainer Benchmark Ultra of Ventana/Roche. Only patients with measurable or evaluable disease, ≥ 18 years, with a WHO performance status of 0–2, and no prior systemic treatment for metastatic or inoperable disease, were considered eligible. Patients with evidence of CNS or leptomeningeal metastases (even if previously treated) or with a history of hematologic or primary solid malignancy (other than TNBC) were excluded (unless in remission for at least 5 years). Inclusion of adequately treated cervical carcinoma in situ or localized non-melanoma skin cancer was permitted. Adjuvant treatment must have been stopped at least 6 months before registration and previous treatment with anthracyclins must not have exceeded 240 mg/m^2^ of doxorubicin or 450 mg/m^2^ of epirubicin.

### Measurements and procedures

At baseline, medical history and symptoms were collected. Additionally, physical examination, blood analysis (hematology, biochemistry, hepatic and renal), pregnancy test, imaging (CT scan thorax-abdomen-pelvis), echocardiography and an ECG were conducted.

For patients without disease progression, imaging was repeated every 3 months. After progression, survival status and documentation of further antineoplastic treatment was conducted every 6 months.

### Intervention

Patients were treated with anti-EGFR-IL loaded with doxorubicin 50 mg/m^2^ iv on day one of a 28-day cycle. Treatment continued until progression. Anti-EGFR targeted nanocontainer drug compounds were produced by inserting anti-EGFR antibody fragments into the membrane of pegylated liposomes (anti-EGFR-ILs-dox) with the payload consisting of doxorubicin, a standard drug for breast cancer, including TNBC. The GMP-compliant preparation was previously described^18^.

### Efficacy and safety endpoints and statistical considerations

The total accrual duration was expected to be 30 months. The minimum follow-up time after the inclusion of the last patient for the primary analysis is 12 months. The primary analysis was time-driven and planned to take place after at least 42 months from start of the trial. The primary endpoint for this trial was progression-free survival at 12 months (PFS12m). PFS was calculated from the date of registration until progression according to RECIST v1.1 or death of any cause, whichever occurred first. Secondary endpoints include: objective response rate (ORR)—defined as proportion of patients achieving CR or PR (RECIST v1.1) during trial treatment; duration of response (DOR)—defined as time from CR or PR (RECIST v1.1) achievement with investigational therapy, until documented progression, relapse, or death due to disease progression; time to progression (TTP)—defined as time from registration until progression (RECIST v1.1) or death due to PD (equivalent to PFS as all deaths were considered as cancer-related); PFS; overall survival (OS)—defined as time from registration until death from any cause; adverse events (AE).

For an alpha = 0.1 with 80% power 47 patients were needed to test the null hypothesis of ≤ 25% of patients to be progression-free at 12 months against the alternative hypothesis of ≥ 40% of patients being progression free at 12 months [with the reference of 25% for the null hypothesis taken from the TNBC subgroup of the ATHENA trial n = 585 TNBC patients^19^. For the primary endpoint, the Kaplan–Meier estimator, one-sided 90% confidence interval (CI) and 95% CI are presented. All efficacy endpoints were analyzed based on the full analysis set (FAS, all registered patients, who received at least one dose of trial treatment excluding patients with major eligibility violations). All safety endpoints were analyzed based on the safety set (all patients, who received any dose of trial treatment). Pre-defined subgroup analyses for all efficacy endpoints were repeated for EGFR + versus EGFR++/+++ patients as defined by immunohistochemistry assessment. All analyses were performed using SAS 9.4 (SAS Institute, Cary, NC, USA) and R 4.0.3 (The R Foundation; www.r-project.org).

### GCP statement

This trial was conducted in compliance with the protocol, the current version of the Declaration of Helsinki^20^, the ICH-GCP^21^ or ISO EN 14155 (as far as applicable) as well as all national legal and regulatory requirements.

### Ethics approval and consent to participate

The study was approved by the ethics committee, EC number 2016-01006, Lead EC Site: Luzerner Kantonsspital Luzern, Switzerland. All participating patients were adults and provided written informed consent.

## Results

### Patients and treatment

Between October 28, 2016 and October 8, 2019, a total of 48 patients were registered. All 48 patients were female with a median age of 59.5 years at time of registration. 9 patients (19%) had previously received doxorubicin whereas 15 patients (31%) had previously received epirubicin in the adjuvant setting. The patients` baseline characteristics can be found in Table 1.

**Table 1:** Patients’ characteristics.

Age at registration (years)	
Median (Min–Max), N = 48	59.5 (28.0–82.0)
WHO performance status	
0	36 (75%)
1	10 (21%)
2	2 (4%)
Body surface area (m^2^)	
Median (Min–Max), N = 48	1.73 (1.35–2.19)
Metastatic at initial diagnosis	20 (42%)
Location of metastases at initial diagnosis*	
Lung	4 (8%)
Liver	6 (13%)
Lymph node	15 (31%)
Bone	9 (19%)
Skin	1 (2%)
Recurrence	
Yes	28 (58%)
No	20 (42%)
Location of metastases at recurrence*	
Breast	13 (27%)
Lung	9 (19%)
Liver	3 (6%)
Lymph node	18 (38%)
Bone	9 (19%)
Other^a^	7 (15%)
Previous treatments	
Treatment with neo-adjuvant chemotherapy	20 (42%)
Treatment with adjuvant chemotherapy	15 (31%)
Previous therapy with doxorubicin	9 (19%)
Previous therapy with epirubicin	15 (31%)
Previous treatment with radiotherapy	26 (54%)
Previous tumor surgery	33 (69%)

Complete cohort of 48 patients.

*More than one location is possible.

^a^Other locations of metastases at recurrence were M. latissimus dorsi right, skin, mastectomy site, pancreas, peritoneal carcinomatosis, peritoneum, skin, soft tissue tumor component in the area of the sternum, chest wall soft tissues.

**Figure 1:**
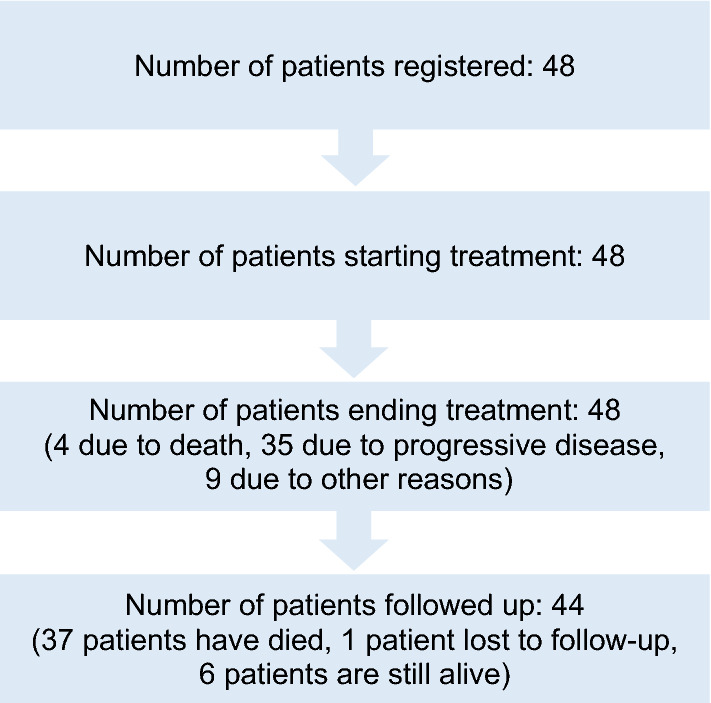
Study flow.

All 48 patients started treatment (consort diagram in Fig. 1). 35 patients stopped treatment due to progressive disease (PD) (72.9%), 4 patients died (8.3%) and 9 (18.8%) patients stopped treatment due to other reasons, such as patient refusal [2 (4%)], start of a treatment not permitted on trial [2 (4%)], unacceptable toxicity [2 (4%)] and withdrawal by the physician [3 (6%)].

The trial was terminated 2 years after the inclusion of the last patient. The last follow-up visit of the last patient included into the trial was 2.2 years after the patient had been included in the trial. A total of 238 cycles had been administered. In 36 cycles (15%) dose reductions were recorded. 16 patients (33%) received at least one cycle with dose reduction, this was due to toxicity in 11 cases (23%), physician’s decision in 2 cases (4%), patient`s decision in 1 case (2%) and other reasons in 3 cases (6%). The median number of cycles was 4 (1 to 21); the median treatment duration was 12.0 weeks (0.1 to 84.6). The median follow-up time was 3.9 years (95% CI [2.6, 4.5]). Up to the time of analysis, 44 patients underwent follow-up, 37 died during this time (84.1%), 6 patients were alive (13.6%). One patient was lost to follow-up (2.3%) (Fig. 1). 8/48 patients (17%) were EGFR + , 40/48 patients (83%) EGFR++/+++ as defined by immunohistochemistry assessment.

### Primary endpoint

Until the time of analysis, 42 events (5 deaths and 37 disease progressions) had occurred and 5 patients were censored due to starting a second line treatment outside of the trial. The Kaplan–Meier estimator for PFS12m was 13% with a one-sided 90% CI of 7%. The corresponding 95% CI is [5%, 25%].

**Figure 2:**
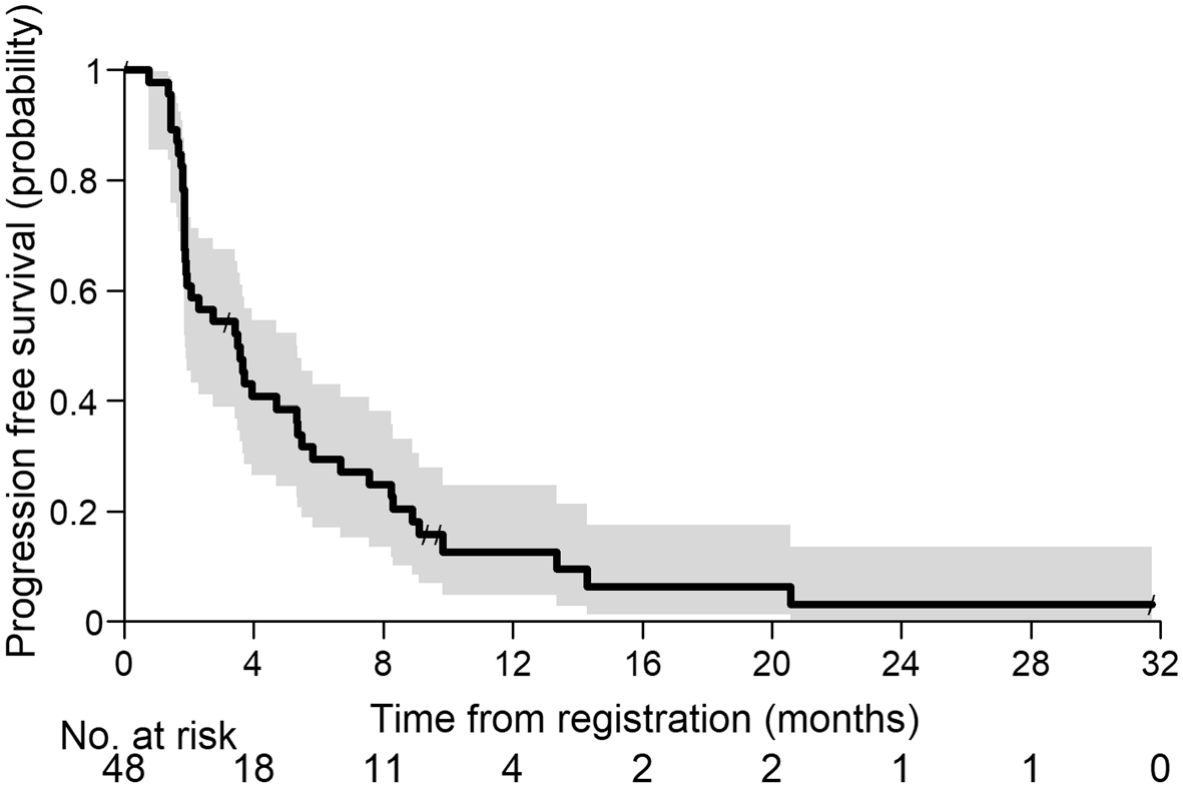
Progression free survival in the full analysis set.

As the lower boundary of the one-sided 90% confidence interval was smaller than 0.25, the null hypothesis cannot be rejected and the primary endpoint was not reached. Median PFS was 3.5 months, 95% CI [1.9, 5.4] (Fig. 2).

**Figure 3:**
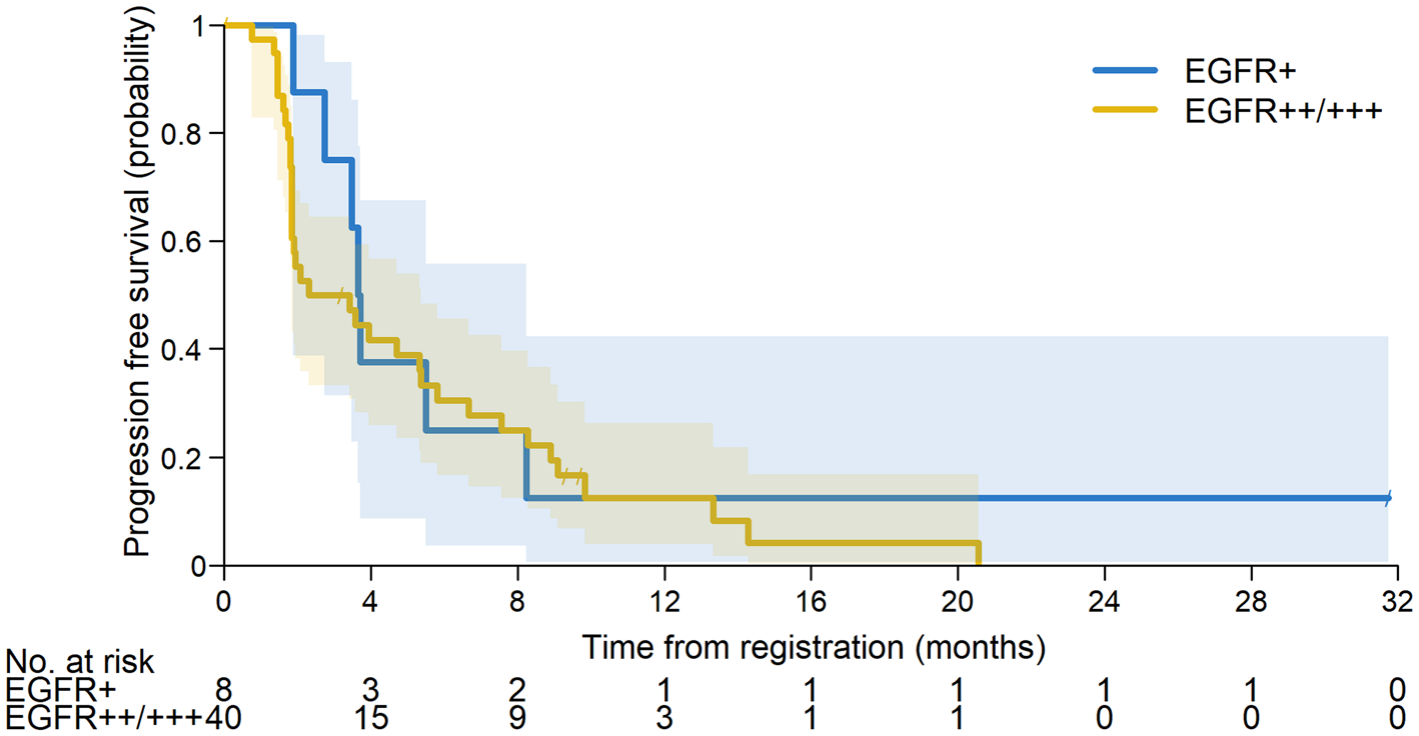
Progression free survival in the full analysis set according to EGFR status.

This was also observed in the subgroup analysis according to EGFR expression: PFS12m was 13%, 95% CI [1%, 42%] in the EGFR + subgroup and 13%, 95% CI [4%, 26%] in the EGFR++/+++ subgroup (Fig. 3).

### Secondary endpoints

The results of the analyses conducted to assess the secondary endpoints are comprised in Table 2.

**Table 2:** Secondary endpoints.

	ORR		DOR		TTP*			OS		
	n	n (%)	N	mo	n	n (%)	mo	n	n (%)	mo
FAS	48	15%, 95% CI [6%, 28%] 0 (0%) CR 7 (15%) PR 19 (40%) SD 16 (33%) PD 6 missing data	7	4.4 months, 95% CI [0.9, 12.6]	48	5 (10%) deceased, 37 (77%) PD	3.5 months, 95% CI [1.9, 5.4]	48	41 (85%) deceased	12.6 months, 95% CI [9.0, 18.0]
EGFR+	8	25%, 95% CI [3%, 65%] 0 (0%) CR 2 (25%) PR 5 (63%) SD 1 (13%) PD	2	2.6 months, 95% CI [0.9, 4.4]	8	7 (88%) PD	3.7 months, 95% CI [1.9, 8.2]	8	7 (88%) deceased	12.9 months, 95% CI [2.5, 24.4]
EGFR++/+++	40	13%, 95% CI [4%, 27%] 0 (0%) CR 5 (13%) PR 14 (35%) SD 15 (38%) PD 6 missing data	5	6.4 months, 95% CI [1.8, 12.6]	40	5 (13%) deceased, 30 (75%) PD	2.9 months, 95% CI [1.8, 5.4]	40	34 (85%) deceased	12.6 months, 95% CI [9.0,19.8]

ORR, overall response rate. CR, complete response. PR, partial response. SD, stable disease. PD, progressive disease. DOR, duration of response. TTP, time to progression. OS, overall survival. FAS, full analysis set.

*Equivalent to PFS as all deaths were considered as cancer-related.

°41 patients have died at time of the current analysis.

Assessment of the ORR showed no complete response (CR). 7/48 patients achieved a partial response (15%), and the majority of patients either had stable disease (SD) or disease progression (PD), regardless of EGFR expression status. Similarly, median OS did not differ between the FAS, the EGFR+ and EGFR++/+++ subgroup.

### Safety evaluation

Over the whole course of therapy, treatment-related grade 1, 2, 3 and 4 AEs were observed in 16.7%, 25.0%, 33.3% and 8.3% of the patients, respectively. 16.7% of patients didn’t develop any treatment-related AEs. Serious AEs (SAEs) occurred in the blood and lymphatic system, the gastrointestinal system, or presented as multiorgan failure, disease progression or affections of the nervous and the vascular system (Table 3). Lower grade adverse events occurred in the blood and lymphatic system, the nervous system, the vascular system, the gastrointestinal and respiratory system, in form of skin changes and immune system disorders, infections/infestations, as elevation of the transaminases and as anorexia and fatigue.

**Table 3:** Serious adverse events.

Adverse events in the FAS (n = 48)				
SOC	Term	Grade 2	Grade 3	Grade 5
Blood and lymphatic system disorders	Febrile neutropenia		1 (2.1%)	
Gastrointestinal disorders	Abdominal pain		1 (2.1%)	
	Mucositis (Small intestinal mucositis)		1 (2.1%)	
	Vomiting	1 (2.1%)		
General disorders and administration site conditions	Multi-organ failure			1 (2.1%)
Neoplasms benign, malignant and unspecified (incl. cysts and polyps)	Progression of disease			1 (2.1%)
Nervous system disorders	Syncope		1 (2.1%)	
Vascular disorders	Thromboembolic event		1 (2.1%)	

Serious adverse events, highest grade as experienced by trial participants.

At the time of the analysis, 41 patients had died, 4 of them during treatment and 37 during the follow-up. 35/41 (85.4%) died from PD, 2/41 (4.9%) died of other (non-drug related) reasons and in 4/41 (9.8%) reason of death was unknown. One trial patient was diagnosed with myelodysplastic syndrome (MDS) about 3.5 years after completing 10 cycles on anti-EGFR-ILs-dox. We interpreted this as a secondary malignancy related to the study drug. The patient started treatment with azacytidine and venetoclax in november 2021. The patient was still alive and the event reported as resolved by the investigator on 10.03.2022. We have no further follow-up information. Otherwise, there were no new toxicity signals compared to the previous phase 1 trial assessing anti-EGFR ILs-dox.

## Discussion

Incorporating technological advances, in particular the use of targeted delivery of potent cytotoxic chemotherapy, is under exploration in the treatment of metastatic TNBC. Especially for antibody drug conjugates, outcome benefits for this patient group have been reported: Sacituzumab govitecan, an antibody–drug conjugate coupling a topoisomerase I inhibitor with a humanized antitrophoblast cell-surface antigen 2 (Trop 2) mAbhRS7 IgG1κ, led to improved PFS and OS in patients with previously treated TNBC when compared to the investigator’s choice of treatment^8^. Despite these advances, there is actually no compound, which promises long-term remission in this hard-to-treat cancer entity.

We have developed anti-EGFR immunoliposomes for the targeted delivery of doxorubicin to EGFR-overexpressing cells. Directed immunoliposomes loaded with a cytotoxic agent follow the same principle as antibody–drug conjugates offering a targeted delivery of a potent drug; however, due to the liposomal encapsulation they alter the tissue distribution and pharmacokinetics of the drug, increasing its therapeutic index, thus providing a unique feature, which could help evolve the landscape of targeted treatments in cancer patients. In addition, the payload carried by an anti-EGFR-ILs nanocarrier is considerably larger than the number of drug molecules used in antibody–drug conjugates. This could potentially further increase the therapeutic efficacy of the compound. In the present trial, we have investigated an anti-EGFR-ILs-dox in a cohort of patients with advanced TNBC. Liposomal encapsulation of doxorubicin and targeted delivery of the compound to EGFR overexpressing breast cancer cells was hypothesized to improve efficacy while at the same time causing less safety signals in comparison to free and untargeted application. This is a novel approach, which, to our knowledge, has not yet been assessed in this entity. While the safety profile of the new compound was acceptable and did not differ from previous results with liposomal doxorubicin, the efficacy of anti-EGFR-ILs-dox in the assessed TNBC population was lower than expected. Thus, this trial did not reach its primary endpoint.

Whether the observed limited efficacy of anti-EGFR-ILs-dox in TNBC is due to the compound itself or to the biology of the disease, remains unclear. In metastatic HER2 + BC a greater efficacy of antibody–drug conjugates depending on the cytotoxic agent was observed, suggesting the importance of the drug delivered by directed therapies^22^. Doxorubicin is a cytotoxic agent classically used in the first line systemic treatment of TNBC, suggesting that the delivered cytotoxic agent used in this trial should be able to elicit a solid treatment response. If we assume that doxorubicin is active, this leads to the question whether EGFR is a suitable target for delivery within this entity. Possibly, tumor heterogeneity might lead to an under- or overestimation of EGFR-expression and thus to a suboptimal delivery of the compound in vivo. In addition, immunohistochemistry as the method of assessment of EGFR expression might not differentiate the biological characteristics, in particular its density on the cell surface and the recirculation of the target in the interior of the cell, with adequate accuracy, thus, making the correlation between EGFR expression and treatment response elusive.

Taken together, there are no signals of efficacy to motivate further development pf anti-EGFR-ILs-dox for treatement of TNBC.

The question arises whether anti-EGFR-ILs-dox would offer more opportunities in other EGFR-expressing malignancies, where targeting EGFR has already shown anticancer effects. This was assessed by our group in glioblastoma (GBM)^23^, where EGFR alterations are found in nearly half of the cases. While treatment with a TKI targeting EGFR has not shown convincing results in GBM patients, some efficacy was reported for an antibody–drug conjugate comprising depatuximab—an IgG1 mAb—coupled to the tubulin inhibitor monomethyl auristatin F (MMAF)^24^. Unfortunately, this could not be confirmed in a first-line trial in combination with temozolomide^25^.

Anti-EGFR-ILs-dox, however, uses immunoliposomes and, thus has a more extensive payload carrying capacity than other drug conjugates. Indeed, we have recently demonstrated that anti-EGFR-ILs-dox can be safely used to deliver cytotoxic molecules to GBM tissue in patients with relapsed GBM (n = 9) harboring an EGFR gene amplification^23^. This may warrant further clinical exploration.

## Conclusions

In summary, this trial investigated the role of anti-EGFR-ILs-dox in TNBC. The main limitation of this study is its one-armed design. We addressed this by using previous data from the ATHENA trial as a comparator, which is, however, a historical data set. Although safe, based on the negative efficacy results of this trial, anti-EGFR-ILs-dox should not be further developed for TNBC. Its potential role in other EGFR-expressing malignancies, such as head and neck cancer and colorectal cancer, will need further assessment in other clinical trials.

## Data Availability

The datasets used and/or analysed during the current study are available from the corresponding author on reasonable request.

## Abbreviations

AE: Adverse Event
Anti-EGFR-IL-dox: Anti-EGFR immunoliposomes loaded with doxorubicin
CI: Confidence Interval
CNS: Central Nervous System
CR: Complete Response
CT: Computed Tomography
DOR: Duration of Response
ECG: Electrocardiogram
EGFR: Epidermal Growth Factor Receptor
ER: Estrogen Receptor
FAS: Full Analysis Set
GMB: Glioblastoma
GMP: Good Manufacturing Practice
HER2: Human Epidermal Growth Factor Receptor 2
mAb: Monoclonal Antibodies
MDS: Myelodysplastic Syndrome
MMF: Monomethyl Auristan F
ORR: Objective Response Rate
OS: Overall Survival
PD: Progressive Disease
PD-L1: Programmed Cell Death Ligand 1
PFS: Progression-Free Survival
PR: Progesteron Receptor
SAE: Serious Adverse Event
SD: Stable Disease
TKI: Tyrosin Kinase Inhibitor
TNBC: Triple Negative Breast Cancer
TTP: Time to Progression
WHO: World Health Organization
